# Highly Toughened Nanostructured Self-Assembled Epoxy-Based Material—Correlation Study between Nanostructured Morphology and Fracture Toughness—Impact Characteristics

**DOI:** 10.3390/polym15071689

**Published:** 2023-03-28

**Authors:** Vasudevan Pillay Remya, Sundararajan Parani, El Hadji Mamour Sakho, Jose Varghese Rajendran, Rodney Maluleke, Thabang Calvin Lebepe, Sam Masha, Nishar Hameed, Sabu Thomas, Oluwatobi Samuel Oluwafemi

**Affiliations:** 1 Department of Chemical Sciences, Doornfontein Campus, University of Johannesburg, Johannesburg 2028, South Africa; 2 Centre for Nanomaterials Science Research, University of Johannesburg, Johannesburg 2028, South Africa; 3 Faculty of Science, Engineering and Technology, Swinburne University of Technology, Hawthorn, VIC 3122, Australia; nisharhameed@swin.edu.au; 4 International and Inter University Centre for Nanoscience and Nanotechnology, School of Chemical Sciences, Mahatma Gandhi University, Kottayam 686560, Kerala, India

**Keywords:** highly toughened, nanostructured, self-assembled, fracture mechanism, impact strength

## Abstract

We present an efficient and effective method for preparing a novel self-assembled nanostructured material with high toughness and impact strength from a blend of di-glycidyl ether of bisphenol-A (DGEBA) and epoxidized poly(styrene-block-butadiene-block-styrene) (eSBS_55_) tri-block copolymer. The field emission scanning electron microscopy and transmission electron microscope results show the nanostructured morphological characteristics of the blends. This study achieved the highest fracture toughness, with a fracture toughness in the form of critical stress intensity factors (*K_IC_*) value of 2.54 MPa m^1/2^, in epoxy/block copolymer blends compared to previous works in the field. The impact strength also increased by 116% compared to neat epoxy. This is a major advancement in epoxy toughening due to the use of a single secondary phase. The resulting highly tough and impact-resistant material is a promising candidate for coating applications in industries such as flooring, building, aerospace, and automobiles.

## 1. Introduction

Toughening of epoxy-based thermoset has seen a remarkable surge in research interest during the past several decades [1,2]. The diversity of approaches, and in particular the addition of a second phase such as elastomers, thermoplastics, block copolymers, and nanoparticles, have made remarkable progress in controlling the morphology of epoxy-based multiphase systems in order to produce highly toughened materials [3,4,5,6,7,8,9]. Out of these research works, the widest attention to producing interesting multiphase morphologies involves the addition of either rubbers [10,11] or thermoplastic materials [12,13,14,15]. However, several disadvantages have been noted, such as a drop in modulus and a decreased glass transition temperature after the addition of rubbers. Furthermore, the maximum recorded fracture toughness value in these systems is less than 1.8 MPa m^1/2^. Hence, over the last 50 years, increasing the fracture toughness of epoxy resins without losing desirable qualities such as modulus and glass transition temperature has been a serious problem [16,17,18].

Self-assembled amphiphilic block copolymers have been used as an effective and intensive modifier to toughen the epoxy resin without sacrificing modulus and glass transition temperature. Amphiphilic block copolymer in epoxy means that one block is “miscible with epoxy” and another block is “immiscible with epoxy. It has been proved that amphiphilic block copolymers have the ability to generate various ordered and disordered nanostructured morphologies via reaction-induced phase separation, and these core-shell nanodomains will be acting as a key factor to fabricate a highly toughened material [19,20,21,22,23,24,25,26,27,28,29,30,31,32,33,34,35,36,37,38,39,40,41,42,43]. This property of amphiphilicity will help in the generation of nanostructured morphology and act as a platform for producing excellent mechanical properties [44,45,46,47]. The major driving force behind the excellent toughness in the epoxy matrix is the compatibility and miscibility of the second phase with the main epoxy matrix, which is achieved by the intermolecular hydrogen bonding with the two phases. This leads to the suppression of macrophase separation and helps to obtain micro phase-separated nano-scale morphology [46,47]. Hillmyer and coworkers studied the formation of nanostructures in epoxy matrix modified using poly(ethylene oxide)-block-poly(ethyl ethylene) (PEO−PEE) and poly(ethylene oxide)-block-poly(ethylene propylene)(PEO−PEP) diblock copolymers [19,20]. Thereafter, several studies have been reported in the same context by varying different types of block copolymers and amphiphilic block copolymers to toughen the epoxy matrix via the development of nanoscale morphology [21,22,23,24,25,26,27,28,29,30,31,32,33,34,35,37,38,39,40,41,42,43,44,45,46,47].

In evaluating the toughening mechanism in epoxy block copolymer blends and hybrid blends, various factors, such as morphology, void growth, plasticization effect, fiber-matrix interaction, rubbery cavitation, etc., have been reported to greatly affect the toughening mechanism with maximum toughness that is below 1.5 MPa m^1/2^ [48,49,50]. In another development, hybrid technique, which combines block copolymer and core-shell rubber mixed with epoxy, has been reported to achieve the highest toughness, although this is less than 2.0 MPa m^1/2^ [50,51]. Hence, it is important to find the most effective epoxy toughening approach and its specific mechanism. A few studies have also revealed the relationship between structure and property of epoxy blends, but there is a broad gap between the correlation study of nanoscale core-shell morphology with the fracture and other mechanical characteristics. To address all these issues, we present a new highly tough self-assembled nanostructured material made from highly epoxidized SBS triblock copolymer (55 mol wt percent) for the first time and explain the relationship between nanostructured morphology and fracture characteristics, as well as possible fracture mechanisms via various factors. 

In our previous studies, we have demonstrated the synthesis of highly epoxidized SBS (55 mol wt%) triblock copolymer and explained the reaction-induced phase separation, nanostructured morphology, wetting studies, viscoelastic, and thermal studies of blends in detail [1,18]. Highly epoxidized SBS block copolymer can enhance compatibility, and it will be a very good additive for producing nanostructured domains. This will significantly affect the critical stress intensity factor (fracture toughness) and impact strength. Our aim is to produce the topmost critical stress intensity factor, *K_IC_* value, that is >2.5 MPa m^1/2^, called highest toughness in epoxy blends. Furthermore, the combination of possible fracture mechanisms such as plasticization, shear yielding, crack deflection, crack pinning, energy dissipation, and others occurred in this system has been explained. We have demonstrated further how the highest epoxidation degree, thickness of rubbery shell, reaction-induced phase separation, and nanostructured core-shell morphology amplify and correlate with fracture toughness and impact characteristics. We believe that the newly synthesized highly toughened material will be a helpful and novel approach towards the development of toughened materials for coating applications such as flooring, various surfaces and woods, vehicles, aircrafts, and other engineering applications.

## 2. Materials and Methods

### 2.1. Materials

The Lapox L12, diglycidyl ether of bisphenol-A (DGEBA) epoxy resin (with an average epoxy equivalent weight of 182–192 gm/eq), Lapox-K5, and 4,4-diaminodiphenylmethane (DDM) were supplied by Atul Ltd. Pala, Kottayam, Kerala, India. The SBS (poly(styrene-block-butadiene-block-styrene) tri block linear copolymer (with a weight average molecular weight of <150,000 gmol^−1^ and the styrene weight percentage of 29.5%) was supplied by Kraton-D1102 AS, Mumbai, India. MCPBA (metachloroperbenzoic acid) and THF (Tetrahydrofuran −99%) were supplied by Sigma-Aldrich, India.

### 2.2. Sample Preparation

#### 2.2.1. Preparation of eSBS_55_

**Scheme 1:**
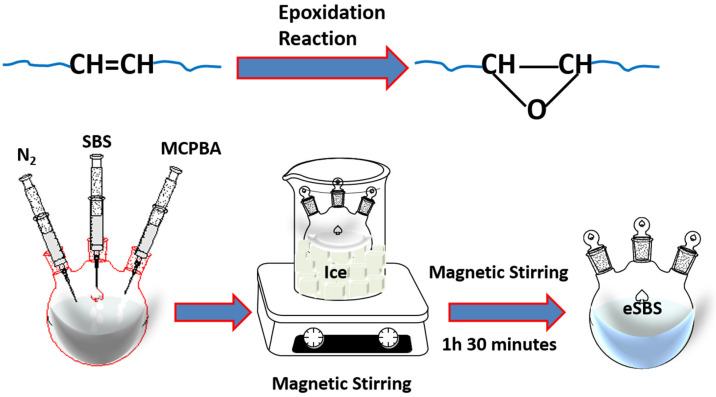
Epoxidation of SBS tri block copolymer.

The epoxidized SBS with a varying epoxidation degree was prepared by using the MCPBA (metachloroperbenzoic acid) method in an inert atmosphere at 0 °C using Hukj as solvent according to our previous report [1,18]. A total of 1 g SBS was dissolved in 70 mL DCM, and stoichiometric amount of MCPBA was added into it. This was followed by magnetic stirring for 1 h 30 min. Scheme 1 represents the epoxidation reaction of SBS block copolymer. The maximum epoxidation degree, 55 mol wt% eSBS, was used for further studies.

#### 2.2.2. Preparation of Epoxy/Epoxidized SBS (55 mol wt%)- 4,4′-diaminodiphenylmethane (epoxy/eSBS_55_-DDM) Blends

The procedure for the preparation for Epoxy/eSBS-0, 5, 10, and 20 phr -DDM blends (phr means parts per hundred) by using the solvent casting method that was described in detail in our previous papers [1,18] In brief, eSBS_55%_ and epoxy resin were dissolved in THF and heated at 80 °C in an oil bath for the complete removal of the solvent. Stoichiometric amount of DDM hardener was added at 85–90 °C under slow magnetic stirring for 10 min. Finally, the mixture was immediately poured into the pre-heated hot silicon mold at room temperature and cured at 90 °C for 3 h and post-cured at 170 °C for 3 h in an oven. The sample was then allowed to cool naturally to room temperature for further studies. 

### 2.3. Characterization Techniques

Transmission Electron Microscopy (TEM): TEM images of epoxy/eSBS_55_-DDM 10 and 20 phr blends were obtained with a JEOL JEM −2100 microscope applying an acceleration voltage of 100 kV. Prior to the analysis, the specimens were prepared using a LEICA Ultracut ultramicrotome. Thin sections of about 70 nm were cut with a diamond knife at room temperature and deposited on a 300 mesh Cu grid, and analyzed without staining. ImageJ software was used to analyze the particle size and distribution.

Fracture Toughness: The fracture toughness tests of neat and epoxy/eSBS_55_-DDM blends were performed by using a Tinius Olsen universal testing machine. The single edge-notched three-point bending test was selected for the evaluation of fracture toughness of the samples. The samples were tested according to standard method ASTM D-5045 with a crosshead speed of 10 mm/min. Rectangular specimens of dimensions 35 × 8 × 4 mm^3^ parallel piped bars were considered, with central V-shaped notches of around 4 mm and a razor-sharp crack tip initiated with a fresh razorblade.

Fracture toughness in the form of critical stress intensity factors (*K_IC_*) was evaluated according to the equation:

(1) $$K_{IC}=\left(\frac{F_{MAX}}{BW^{\frac{1}{2}}}\right)f\left(\frac{a}{W}\right)$$

where ‘*F_max_*’ is the maximum load at failure, ‘*W*’ is the width of the specimen, ‘*B*’ is the thickness of the specimen, ‘*a*’ is the cracklength, and *f*(*a*/*W*) is the factor of geometry described in ASTM standard D-5045.

Izod Impact Test: The Izod impact strength of the neat and epoxy/eSBS_55_-DDM blends was analyzed at room temperature for notched specimens with the dimensions of 63.5 × 12.7 × 3 mm as indicated in ASTMD256 using a ZwickRoel HIT25P impact tester machine having 5.5 J at 23 °C as per ASTM D 256.

Field Emission Scanning Electron Microscopy (FE-SEM): FE-SEM micrographs of epoxy/eSBS_55-_DDM blends were taken in a Nova Nano FESEM 450 UoK Backscattered Electron (BSE) mode by applying 5.00 kV accelerating voltage and field-free lens mode. The samples were treated with liquid nitrogen to make them fragile and became easily breakable by hand. A small portion of the sample was then taken for analysis.

## 3. Results

### 3.1. Evolution of Nanostructured Core-Shell Morphology

The addition of the highest epoxidized SBS (55 mol wt%) that is compatible with epoxy resin leads to viscoelastic phase separation and plasticization effect. The detailed mechanism behind the generation of nanostructured core-shell morphology in epoxy/eSBS55 blend is explained in our previous studies [1,18]. Briefly, when we add 10 phr and 20 phr eSBS55 in epoxy, a homogeneous system appeared initially and later the homogeneity of the system disappeared. This is due to the reaction-induced phase separation and unfavorable interactions. Due to the nonpolar–nonpolar interactions, the unmodified PB block self-assembles around the phase separated PS and forms spherical micelles in the intermediate stage. The process of nucleation and growth followed by self-assembly begins, resulting in the creation of core-shell nanodomains with PS as core and PB as shell. George et al. in the epoxy block copolymer blend method recorded the same kind of phase separation and morphologies [52,53,54].

**Figure 1:**
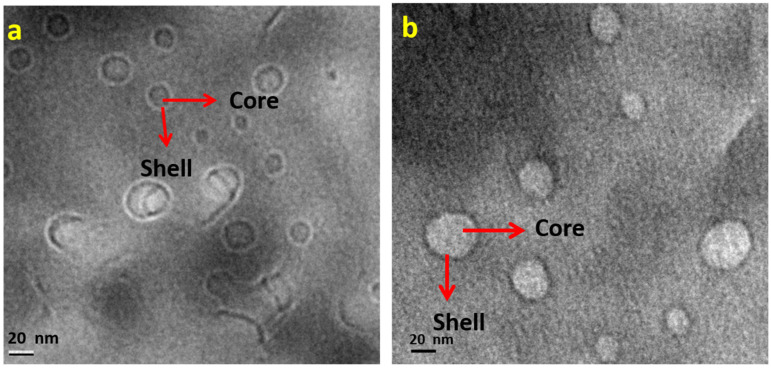
HR-TEM images of (**a**) 10 phr and (**b**) 20 phr epoxy/eSBS_55_ blends.

To design a highly tough material by using epoxy, initial requirement is the choice of block copolymer and its good compatibility with epoxy resin. In this study, eSBS_55_ block copolymer has fulfilled these criteria for selective compatibilization of blocks in epoxy and led to reaction-induced phase separation (RIPS). This reaction-induced phase separation leads to nucleation and growth (NG) and self-assembly process, which play a major role in creating nanostructured morphology via microphase separation and offer an avenue for excellent mechanical properties in epoxy thermoset. The TEM images in Figure 1 prove the formation of nanostructured core-shell morphology. In Figure 1, it can be seen that the continuous phase of epoxy in which epoxidized SBS is spread accounts for the majority of the brighter regions in the image. When eSBS mixed with epoxy resin, the unmodified poly butadiene-PB and poly styrene-PS parts become phase-separated out of the homogenous continuous epoxy phase, forming nanodomains with a spherical morphology. The particle size of the core/shell upon the addition of 10 phr and 20 phr blends was calculated to be 13–30 nm and 16–40 nm, respectively. Another intriguing component is the nanodomain shell thickness in the 10 phr blend, which is 11 nm, and in the 20 phr blend, it is 6 nm. The thick rubbery shell helps to produce more visco-elastic properties, and this will lead to reaction-induced phase separation and induce more toughness [1]. Hence, this thick rubbery shell (PB), critical domain size and good dispersion of particles in the 10 phr blend can absorb more fracture energy, which is a notable result in our study for achieving high toughness compared to other documented epoxy-toughening work. However, in the 20 phr blend system, a viscous nature was observed, and the presence of agglomerates was noted. Furthermore, no evidence of fine particle distribution was detected. In short, the core-shell nanostructured morphology that has developed in 10 and 20 phr blends clearly suggests and correlates with outstanding mechanical properties. Our previous paper also discussed the TEM images of epoxy/eSBS blends including 5 phr and 10 phr in detail [1].

**Scheme 2:**
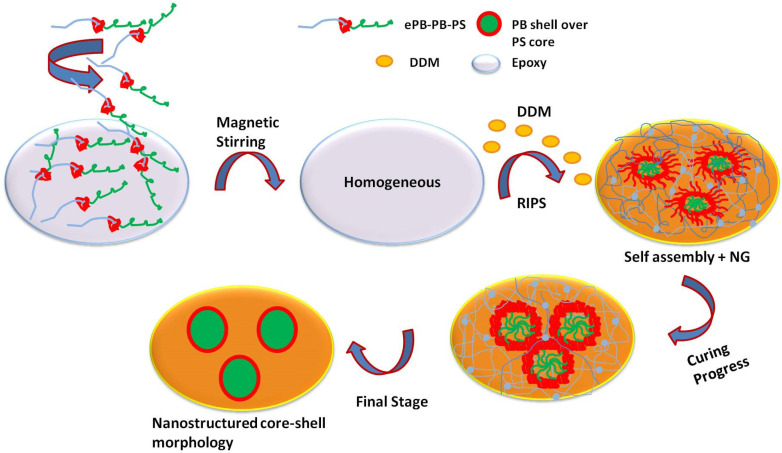
Schematic representation of the formation of nanostructured core-sell morphology in epoxy/eSBS_55_ blends system.

The above-mentioned mechanisms and evolution of the highly ordered nanostructured spherical core-shell morphology system are clearly depicted in the schematic representation in Scheme 2.

### 3.2. Mechanical Studies

The toughness measurements and impact properties are the major mechanical properties of epoxy resin. Recent toughening strategies in epoxy include the addition of block copolymer alone, mixed block copolymers, and a hybrid approach combining block copolymer with rubber or nanoparticles, among others. Perez et al. claim to have discovered a toughening process in epoxy using the PEP-PEO/PS-PEO modifier [48]. Dean et al. demonstrated that the vesicle morphologies created in the brominated and nanobrominated epoxy resin/PEO-PEP/(PMMA-ran-GMA-PEHMA) system are more tough than the spherical and wormlike morphologies [49]. Larger vesicle diameters resulted in a three-fold increase in fracture resistance. Klinger et al. attempted to improve toughness in epoxy by employing a hybrid technique, namely a block copolymer-core shell rubber hybrid toughening [50]. They achieved a maximum *K_IC_* value of 1.4 MPa m^1/2^ in their study. Despite employing a hybrid technique in their system, they are unable to achieve high toughness. Functionalized block copolymers can offer better toughness than any other combination, according to current research. George et al. have reported excellent fracture toughness and impact strength by varying epoxidation degree in SBS block copolymer and reported maximum fracture toughness value (1.74 MPa m^1/2^) in 20 phr epoxy/eSBS blends with 47 mol wt% epoxidation degree [52,53]. As a result, the epoxidized SBS block copolymer behaves like an amphiphilic in nature and has been used as an efficient epoxy modifier. As a result, we used the MCPBA method to obtain a greater epoxidation degree in SBS block copolymer by altering various parameters, and, for the first time, we were able to achieve a maximum of 55 mol wt% percent epoxidation degree in SBS block copolymer (as stated in detail in our prior study). On evaluating all these reported studies critically, our present attempt is to significantly improve the mechanical properties. We have formulated the highest epoxidation degree, nanostructured core-shell morphology, RIPS, and high rubbery shell thickness by altering cure time, cure temperature, and other processing parameters.

#### 3.2.1. Fracture Toughness

**Figure 2:**
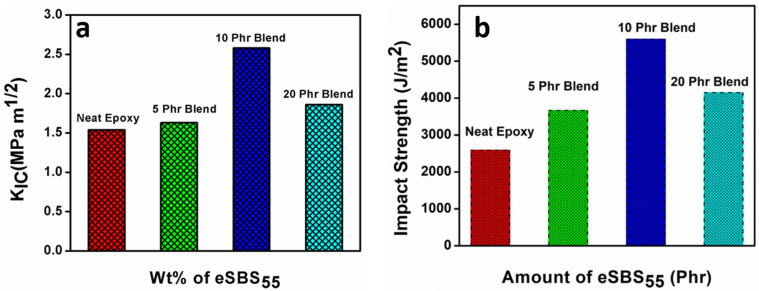
(**a**) Fracture toughness and (**b**) impact strength of neat epoxy and epoxy/eSBS_55_ blends systems.

The effects of various concentrations of eSBS with 55 mol wt% on the fracture toughness of the epoxy blends were investigated (Figure 2a). Increasing the concentration of eSBS_55_ content increased the toughness gradually. The most remarkable and highest improvement came with the blend with 10 phr eSBS_55_. In this work, fracture toughness values were measured as a critical stress intensity factor, ‘*K_IC_*’, and the topmost value obtained in 10 phr blend was 2.54 MPa m^1/2^**.** It is noteworthy that other research works in epoxy with amphiphilic block copolymers and other second phase inclusions only reported maximum of 2.2 MPa m^1/2^ toughness value (*K_IC_*) [50,52,53,54]. 

The fracture toughness and other mechanical properties of the epoxy/eSBS_55_ blend system also correlate with nanostructured core-shell morphology. Neat epoxy is transparent and its ‘*K_IC_*’ is 1.54 MPa m^1/2^. The addition of unmodified SBS lowered the toughness value of epoxy and showed opaqueness because of macrophase separation and poor interfacial interaction between epoxy and SBS [52,53]. The *K_IC_* value increased gradually from 1.54 MPa m^1/2^ (epoxy) to 2.54 MPa m^1/2^ in 10 phr blend via 1.62 MPa m^1/2^ (5 phr blend). This is due to the change in morphology from the microlevel to nanolevel (Supplementary Materials, Figure S1). This increase in *K_IC_* value significantly affects the enhancement in the critical stress intensity factor. Specifically, the phase-separated particles in a 10 phr blend have a shell thickness of 11 nm and a size range of 13 to 30 nm. The detailed mechanism of reaction-induced phase separation was explained in the previous Section 3.1. This thick rubbery shell (PB), critical domain size, and good particle dispersion in the 10 phr blend can absorb more fracture energy, which is an important finding in our study to obtain highest toughness in comparison to other reported epoxy toughening studies. The phase-separated domains in the 20 phr blend, however, reveal a low shell thickness of 6 nm and a size range of 16 to 40 nm. In addition, the 20 phr mix system did not have fine particle dispersion; it had a viscous character, and agglomeration was apparent. Therefore, on top of the addition of more block copolymers (20 phr), the fracture toughness gradually decreases to 1.86 MPa m^1/2^. This is due to the high viscosity of the system, increased size of phase domains, and thin rubbery shell thickness. Morphological studies show that there is a transition between the rough fracture surface and the smooth fracture surface when adding a second high phr phase into epoxy systems. Therefore, the toughness of the blend system gradually decreases with high phr addition of block copolymers to it. Similar types of explanations for the high phr addition of block copolymer in epoxy have been reported by George et al. and Parameswaranpillai et al. This low toughness of 20 phr blend will result in high viscosity and a reduction in toughness rating. Additionally, these factors were thoroughly covered in our earlier investigations [1,4,18,52,54].

**Figure 3:**
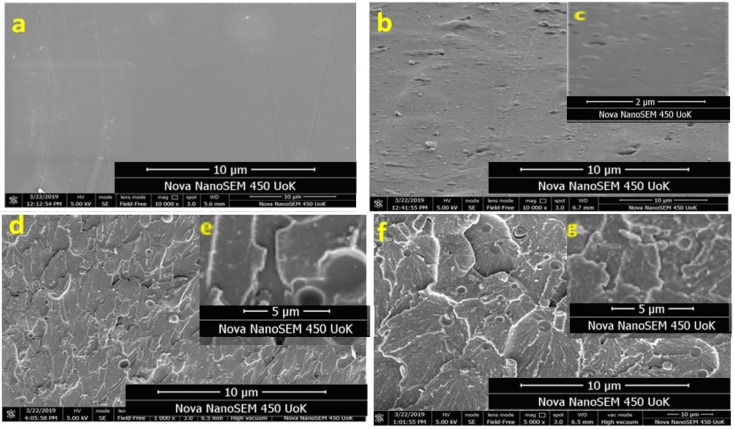
FE-SEM micrographs of fracture surface of (**a**) neat epoxy, epoxy/eSBS_55_blends with (**b**) 5 phr, (**c**) higher magnification, (**d**) 10 phr, (**e**) higher magnification, (**f**) 20 phr, and (g) higher magnification, respectively.

**Scheme 3:**
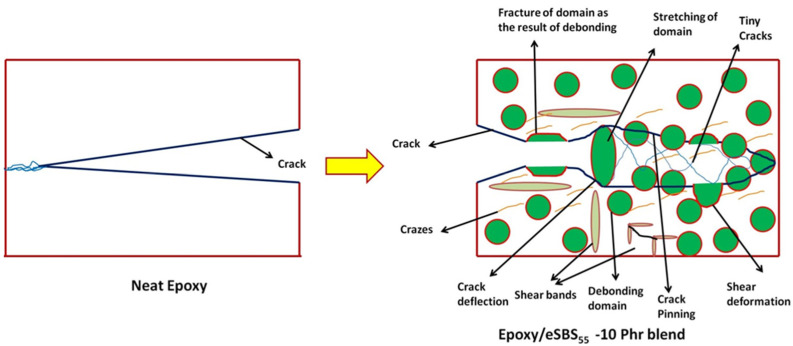
A schematic representation of possible mechanism by which eSBS55 toughens epoxy.

We noticed that the neat epoxy system exhibits smooth morphology, and large cracks are propagated regularly and freely and also oriented in the direction of load in SEM images (Figure 3a). Therefore, the cracks propagate easily, and the material breaks catastrophically like brittle material under the influence of even a small load (0.75 MPa m^1/2^). A careful examination of 10 phr and 20 phr blends shows that the nano domains are uniformly distributed in the continuous epoxy matrix. The hydrogen bonding interactions between the hydroxyl groups of the growing epoxy matrix and epoxy groups of the epoxidized block are more pronounced in the 10 phr and 20 phr blends and lead to good adhesion between the epoxy and nano dispersed domains. This factor is a key point for the highest improvement in critical stress intensity factor. Hence, the load applied is effectively transferred to all nanodomains from the major plane of epoxy phase. From the following SEM images, (Figure 3b–e), we have also observed rough morphology and so many tiny cracks oriented in all directions and deviated from the original plane. This will increase the surface area of the cracks, leading to tremendous improvement in fracture toughness. That is to say, the load is transferred to each nanodomain, and the pinning of cracks by nanodomains and crack deflection take place. Therefore, crack pinning and deflections are the major fracture mechanisms observed (Scheme 3 and Figure 3). Nanodomains have the ability to initiate an energy-absorbing mechanism, and this will also cause an increase in critical stress intensity factor [50,51,52,54]. Additionally, we believe that nanoscale cavitation of the rubbery shell and its plastic deformation contribute to the improvement in toughness of epoxy/eSBS blends. In addition, the rubbery shell micelle outperforms its glassy core equivalent and overcomes toughening mechanism deficiencies. The higher thickness of rubbery shell amplifies the reduction of crosslinking density and altered shear yielding. The plasticization effect, nanocavitation, and voids filling through nanodomains can create network disruption, which can endure higher energy transfer and result in increased toughness. The crosslinking density of epoxy is reduced by the addition of eSBS due to plasticization effect. This is another positive factor leading to the enhancement in toughness. In summary, the toughening mechanism of eSBS_55_-modified epoxies with nanostructured spherical core-shell morphology could be attributed to the combined effect of crack pinning, crack deflection, shear yielding, nanocavitation, energy dissipation, plastic deformation, interfacial debonding of spherical domains, and matrix void expansion (Scheme 3). This level of combination is uncommon in epoxy-based systems, and we would like to add further that this was made possible due to the high epoxidation degree. 

#### 3.2.2. Impact Strength

Figure 2b represents the pendulum impact test results of neat epoxy and epoxy/eSBS_55_ blends with different concentrations of eSBS_55_. The results show that the impact strength of neat epoxy has been significantly improved by the addition of eSBS_55_ and showed maximum value at 10 phr blend. Neat epoxy showed the impact strength value of 2590 J/m^2^, and after adding 5 phr eSBS_55_ block copolymer, a 41% improvement in impact strength was observed. The impact strength was enhanced effectively in a 10 phr blend, which shows a 116% increase in impact strength as compared to neat epoxy system. Further addition of epoxidized block copolymer decreases the impact strength. This could be attributed to the inefficient dispersion of eSBS_55_ as a result of the high viscosity. The 20 phr blend showed an impact strength of 4149 J/m^2^, which is 60% higher than the impact strength of neat epoxy. Impact strength and impact energy values of neat epoxy and epoxy/eSBS_55_ block copolymer blends with various concentrations are shown in Table 1. As a result of the energy-absorbing mechanism that nanodomains can start, the critical stress intensity factor will rise [50,51,52,54]. Additionally, we think that the rubbery shell’s plastic deformation and nanoscale cavitation play a role in the increased toughness of epoxy/eSBS composites. Moreover, the rubbery shell microcelle surpasses its glassy core equivalent and bypasses weaknesses in the toughening mechanism. The reduced crosslinking density and changed shear yielding are amplified by the thicker rubbery shell. Network disruption can be caused by the plasticization effect, nanocavitation, and voids filling through nanodomains. These phenomena can withstand higher energy transfer and increase impact strength. Due to the plasticization effect, the inclusion of eSBS reduces the crosslinking density of epoxy. That is to say, the reason behind the high impact values is the same as that of fracture toughness and are explained in detail in the previous section and Scheme 3. As explained earlier, multiple mechanisms operate that include the crack pinning, crack deflection, shear yielding, nanocavitation, energy dissipation by plastic deformation, interfacial debonding of nano domains, and matrix void expansion. 

### 3.3. Field Emission Scanning Electron Microscopy (FE-SEM)

Figure 3 shows the fracture surface FE-SEM images of cured epoxy and epoxy/eSBS_55_-5, 10, and 20 phr blends. The fracture image of cured neat epoxy in Figure 3a shows a single phase, flat, homogeneous, smooth, freely, and regularly oriented cracks in the direction of load. This indicates the glassy and brittle nature of the neat epoxy resin. In the 5 phr blend (Figure 3b,c), the homogeneity disappeared and microscopic phase separation occurred. The eSBS particles are uniformly dispersed in the epoxy matrix and forms droplet–matrix morphology. This blend is also transparent, indicating the absence of macrophase separation. An interesting morphological change as a function of eSBS content is observed in epoxy/eSBS_55_-10 and 20 phr blends (Figure 3d–g). The 10 phr blend shows a very rough, heterogeneous, and flakes-like morphology, while Colocasia leaf-like morphology was observed in 20 phr. This system is very rough and heterogeneous. In both 10 and 20 phr blends, initially, the soft eSBS part is easily miscible with epoxy resin. Later, because of reaction-induced phase separation upon curing and polymerization of epoxy, the PS part of the block copolymer phase-separated and dispersed as spherical globular nano domains in the continuous epoxy matrix. The domain size also in the nanoscopic level in 10 and 20 phr blends because of the good compatibility of eSBS in the epoxy matrix. Care examination of the fracture surfaces clearly indicate crack pinning, crack deflection, shear yielding, nanocavitation, and interfacial debonding of nano domains. 

## 4. Conclusions

In this study, we have successfully fabricated a highly toughened self-assembled nanostructured material in an epoxy thermoset by incorporating an amphiphilic block copolymer, the highly epoxidized SBS triblock copolymer, through the solvent casting method. TEM microscopy studies showed the formation of well-ordered core-shell spherical nanodomain morphologies in the epoxy/eSBS55 blends. The 10 phr blend exhibited exceptional improvement in impact strength and fracture toughness, with a value of 5597 J/m^2^ for impact strength, a 116% increase compared to the neat epoxy system. The high toughness was attributed to various mechanisms, such as crack pinning, crack deflection, shear yielding, nanocavitation, energy dissipation by plastic deformation, inter-facial debonding of nano domains, and matrix void expansion. The results of our study demonstrate that the highly epoxidized SBS block copolymer is an effective modifier for producing a super-toughened and high impact strength material in epoxy systems.

## Figures and Tables

**Table 1:** Impact strength and impact energy values of neat epoxy and epoxy/eSBS blends.

Specimen	Impact Strength (J/m^2^)	Impact Energy (J/m)	Fracture Toughness *K_IC_* (MPa m^1/2^)
Neat Epoxy	2590 ± 42	27 ± 3	1.54
Epoxy/eSBS-5 phr blend	3668 ± 56	36 ± 5	1.62
Epoxy/eSBS-10 phr blend	5597 ± 62	58 ± 6	2.54
Epoxy/eSBS-20 phr blend	4149 ± 34	43 ± 5	1.86

## Data Availability

The raw/processed data required to reproduce these findings cannot be shared at this time as the data also form part of an ongoing study.

## Supplementary Materials

The following supporting information can be downloaded at: https://www.mdpi.com/article/10.3390/polym15071689/s1, Figure S1: Microlevel to nano level morphology change. (5 to 10 Phr transition); Figure S2: Transparency of Images; Table S1: Data for E1g, E1r and Tg of the developed material.
