# Supplementary material for: Highly Toughened Nanostructured Self-Assembled Epoxy-Based Material—Correlation Study between Nanostructured Morphology and Fracture Toughness—Impact Characteristics
Source: Polymers (Basel). 2023 Mar 28;15(7):1689. doi: 10.3390/polym15071689 (PMC10097291; doi:10.3390/polym15071689)
Supplement: Supplementary file 1 [file polymers-15-01689-s001.zip › polymers-2162903-supplementary.pdf]

Supporting Document

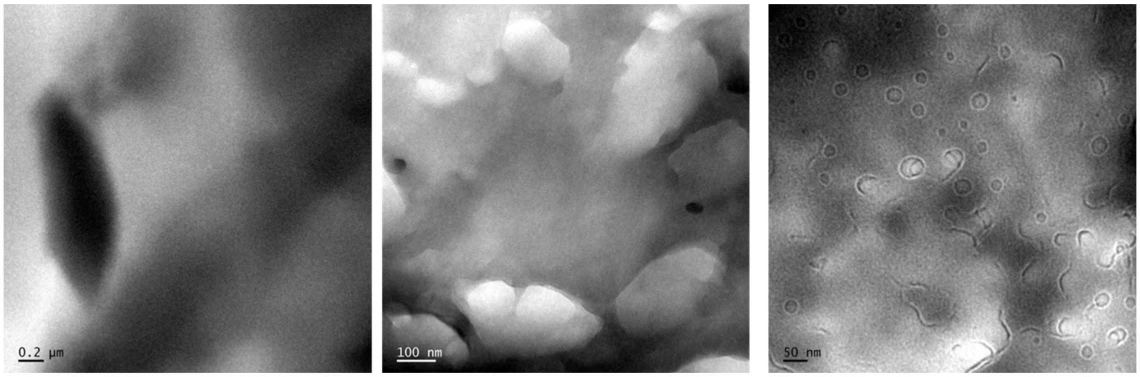

Figure S1. Microlevel to nano level morphology change. (5 to 10 Phr transition).

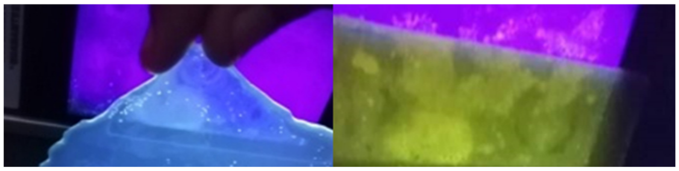

Figure S2. Transparency of Images (Neat epoxy-blue and cured epoxy/$eSBS_{55}$-10 phr blend-Yellow).

Table S1. Data for $E^{1}g$, $E^{1}r$ and T_g of the developed material.

Sample	Storage Modulus in the Glassy region ($E^{1}g$) MPa	Storage Modulus in the Rubbery region ($E^{1}r$) MPa	T_g (°C)
Neat epoxy	1880	86.11	172
Epoxy/eSBS-10 phr	2854	92.27	153

The glass transition temperature of neat epoxy lowered as the addition of second phase to it because of viscoelastic phase separation and RIPS. Storage modulus value increases as the addition of %%% epoxidized SBS into neat epoxy system.
